# A Volumetric Absorptive Microsampling Technique to Monitor Cannabidiol Levels in Epilepsy Patients

**DOI:** 10.3389/fphar.2020.582286

**Published:** 2020-11-16

**Authors:** Sara Dubois, Francesca Marchese, Federica Pigliasco, Sebastiano Barco, Gino Tripodi, Tommaso Lomonaco, Simona Lattanzi, Emilio Russo, Giuliana Cangemi, Pasquale Striano

**Affiliations:** ^1^ Department of Neurosciences, Rehabilitation, Ophthalmology, Genetics, Maternal and Child Health, University of Genoa, Genoa, Italy; ^2^ Pediatric Neurology and Muscular Diseases Unit, IRCCS Istituto Giannina Gaslini, Genoa, Italy; ^3^ Chromatography and Mass Spectrometry Section, Central Laboratory of Analyses, IRCCS Istituto Giannina Gaslini, Genoa, Italy; ^4^ Department of Chemistry and Industrial Chemistry, University of Pisa, Pisa, Italy; ^5^ Neurological Clinic, Department of Experimental and Clinical Medicine, Marche Polytechnic University, Ancona, Italy; ^6^ Science of Health Department, School of Medicine, University Magna Graecia, Catanzaro, Italy

**Keywords:** epilepsy, therapy, cannabidiol, therapeutic drug monitoring, volumetric absorptive microsampling, refractory seizures

## Abstract

**Purpose:** Interest in cannabis-based therapies has recently increased, due to the availability of cannabidiol (CBD) for the treatment of epilepsy without psychoactive effects. Therapeutic drug monitoring can prevent drug interactions and minimize drug toxicity. We evaluated a volumetric absorptive microsampling (VAMS) method combined with LC-MS/MS (liquid chromatography coupled to tandem mass spectrometry) for the quantification of CBD blood levels in patients with refractory epilepsy.

**Methods: **Prospective observation of patients with Dravet syndrome receiving open-label, add-on GW-purified CBD (Epidyolex^®^) at different doses. CBD plasma samples were obtained from venipuncture and LC-MS/MS was used to measure CBD in venous and capillary blood samples collected by VAMS.

**Results:** We enrolled five patients with a mean age of 13 (range: 4–27) years. CBD levels measured by VAMS on capillary blood did not differ from CBD levels measured in plasma by venipuncture (*R* ^2^ > 0.93).

**Conclusion:** This proof-of-concept study suggests that VAMS allows monitoring of CBD plasma levels and can offer valuable support for personalized therapy in refractory epilepsy.

## Introduction

Epilepsy is one of the most common brain chronic disorders, affecting around 70 million people of all ages worldwide (Hirtz et al., 2007; Zaccara and Schmidt, 2017). The identification of the appropriate treatment allows in most patients a medium and long-term remission in seizures control (Striano and Striano, 2009; Striano et al., 2016; Lattanzi et al., 2019). Despite the use of numerous therapeutic options, including third-generation antiseizure medications (ASMs), neuromodulation, surgical and dietary interventions, 30% of patients continue to have seizures (Striano and Striano, 2009; Zaccara and Schmidt, 2017).

The interest in cannabis-based therapies has increased, in particular in the two main phytocannabinoids: cannabidiol (CBD) and Δ9-tetrahydrocannabinol (THC). CBD stimulates interest because of its anti-convulsive properties in absence of psychoactive effects and abuse liability, unlike THC (Devinsky et al., 2014; Arzimanoglou et al., 2020). The therapeutic potential of galenic preparations marketed to contain CBD/THC was found to depend on preparation procedures, components concentration, and presence of other constituents (De Caro et al., 2017; Carcieri et al., 2018; Lattanzi et al., 2018; Lattanzi et al., 2019). Purified CBD produced by GW pharma (EPIDYOLEX^®^) is the first of a new class of ASMs. (Lattanzi et al., 2019; Lattanzi et al., 2020). The approval in July 2019 by the European Medicines Agency to use CBD as an additional treatment with clobazam in two forms of childhood refractory epilepsy (Dravet syndrome and Lennox-Gastaut syndrome) is a milestone in the medical use of phytocannabinoids for the treatment of epileptic disorders. Due to the heterogeneity of epilepsy clinical manifestations and interindividual response to old and new antiepileptic drugs, therapeutic drug monitoring (TDM) is a valuable clinical support in patients’ treatment.

In refractory epilepsy, the relationship between the dose administered and CBD blood levels demonstrated in some studies (Geffrey et al., 2015; Landmark and Brandl, 2020) has provided a starting point for the use of TDM in the wide variability of CBD pharmacokinetics (Ocque et al., 2019). TDM is useful in clinical practice as it allows to obtain the ideal dose of cannabis-based therapy based on the identification of the individual concentration associated with an optimal response. Moreover, in polypharmacy TDM can prevent drug interactions by guiding dose adjustments and minimizing toxicity (Striano et al., 2008; Patsalos et al., 2018; Brandt, 2019). Microsampling techniques based on dried blood spots allow a reliable and non-invasive collection of small blood volumes. Recently, the novel device VAMS (Volumetric Absorptive Microsampling) has been introduced in the market, commercial name MITRA^®^, successfully applied to several quantitative TDM methods. This device allows the collection of a fixed volume of blood (10 or 30 µl) avoiding the effect of hematocrit (HCT) on the analytical performances (Mano et al., 2015; Barco et al., 2017; Kok and Fillet, 2018; DʼUrso et al., 2019). We evaluated VAMS in combination with liquid chromatography coupled to tandem mass spectrometry (LC-MS/MS) for the quantification of CBD blood levels to be used in clinical practice to personalize the cannabis-based treatment of refractory epilepsy. In particular, we determined CBD concentrations in capillary and venous blood obtained by micro-sampling and compared them with CBD concentration in plasma, which is the matrix most frequently used for TDM in epilepsy patients.

## Methods

### Participants

We investigated five subjects with Dravet syndrome treated with CBD oral solution (Epidyolex^®^) given for compassionate use. All participants were taking a stable dose of ASMs and were followed-up prospectively through medical charts and parents/caregivers’ information.

### Study Design

Patients received Epidyolex as compassionate use approved by the Regional Ethics Committee. Written informed consent was signed by parents, caregivers, or legal representatives. CBD was administered at the initial dose of 2.5 mg/kg two times per day (5 mg/kg/day) to be increased after 1 week to a maintenance dosage of 5 mg/kg twice daily (10 mg/kg/day). The CBD dose could be increased in weekly increments of 2.5 mg/kg twice daily according to clinical response. Physical examination and laboratory tests (FBC, serum sodium, potassium, chloride, creatinine, ALT, AST, total bilirubin, INR, and glucose) were performed at baseline (within 2 weeks after initiation of CBD treatment) and after 1 month, 3 months, and 6 months of treatment. Patients’ parameters, i.e., weight, height, and body mass index, were recorded at each scheduled visit and a safety check was carried out by monitoring CBD plasma levels by venipuncture. CBD blood levels were evaluated at least 3 months after the start of treatment. During the monitoring study of the different cannabis-based therapies, the doses of concomitant ASMs administered to patients were not modified, establishing an appropriate observation condition.

### Quantification of Cannabidiol in Plasma by Volumetric Absorptive Microsampling-Liquid Chromatography Coupled to Tandem Mass Spectrometry

Blood samples were obtained in the morning before the first daily medication. Venous blood was collected by venipuncture on tubes containing ethylenediaminetetraacetic acid and plasma was separated by centrifugation at 2,000 *g* for 5 min. The 30 µl VAMS devices (MITRA^®^, Neoteryx, Torrance, CA, United States) were used to collect venous and capillary blood. The venous VAMS samples were obtained from blood collected by ethylenediaminetetraacetic acid tubes, as described (Barco et al., 2018; Pigliasco et al., 2020). Capillary VAMS were obtained following the manufacturer’s instructions: before pricking the patient’s finger with a microneedle, the area was disinfected and after the first drop of blood was removed, the VAMS tip was placed in contact with the surface of the second drop to adsorb the matrix.

### Statistical Analysis

The correlation between CBD venous and capillary VAMS and CBD plasma levels was assessed by linear regression analysis (“Medcalc,” Software Ltd., Ostend, Belgium).

## Results

### Demographic Characteristics and Compliance

The demographic and clinical features of the participants are summarized in Table 1. Four subjects were males and the mean age of the five patients was 13 (range: 4–27) years. At enrollment, all participants had failed from two to four ASMs and were on stable treatment (mean: three concomitant drugs) for at least 3 months before CBD add-on. Table 1 also shows the dose and amount of CBD provided to each subject. The mean dose of Epidyolex administered was 658 mg/day (15 mg/kg/day).

**TABLE 1:** Demographic and clinical features of the patients.

Patient/Age (years)	BMI (kg/m^2^)	Total dose (mg/day)	Fraction (mg/day)	Concomitant antiseizure medications
#1/(4–6)	19.6	385 (17.5 mg/kg/day)	192 mg × 2 days	Valproic acid (378 mg/kg/day), stiripentol (750 mg/kg/day), clobazam (10 mg/kg/day), topiramate (100 mg/kg/day)
#2/(10–12)	28.1	650 (10 mg/kg/day)	325 mg × 2 days	Valproic acid (1,000 mg/kg/day), clobazam (15 mg/kg/day), levetiracetam (16 mg/kg/day)
#3/(16–18)	13.5	665 (17.5 mg/kg/day)	332 mg × 2 days	Valproic acid (650 mg/kg/day), stiripentol (1,500 mg/kg/day), topiramate (100 mg/kg/day)
#4/(25–27)	22.5	1,360 (20 mg/kg/day)	680 mg × 2 days	Valproic acid (600 mg/kg/day), stiripentol (1,500 mg/kg/day), clobazam (10 mg/kg/day), topiramate (200 mg/kg/day)
#5/(7–9)	14.7	230 (10 mg/kg/day)	115 mg × 2 days	Valproic acid (500 mg/kg/day), stiripentol (500 mg/kg/day)

### Outcome Therapeutic Monitoring Cannabidiol Levels by Venipuncture and Volumetric Absorptive Microsampling

**FIGURE 1:**
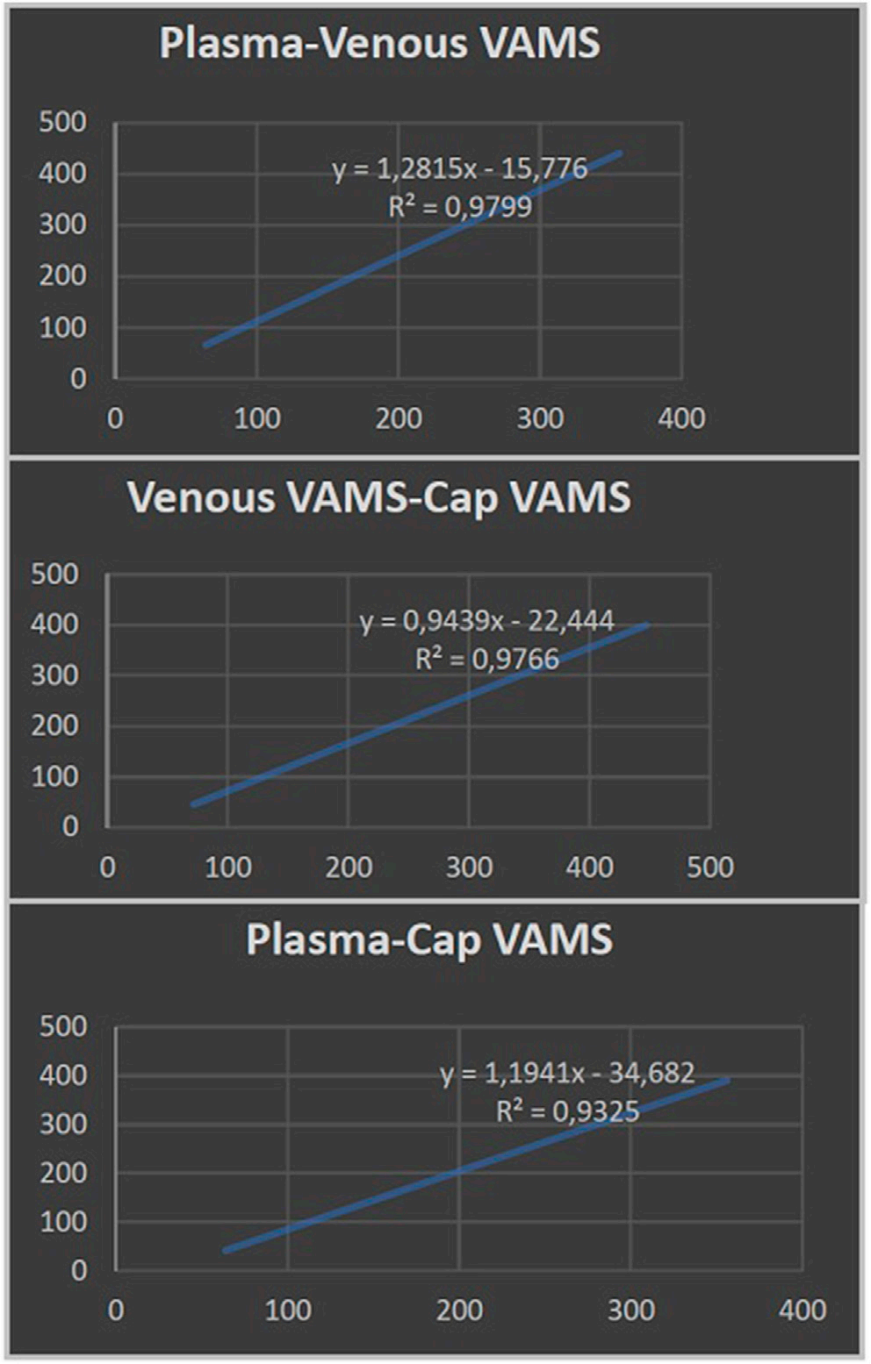
Linear regression analysis showing the correlation between cannabidiol plasma levels from venipuncture and cannabidiol concentrations measured by venous and capillary volumetric absorptive microsampling (VAMS).

The results achieved from the analysis of plasma and venous and capillary VAMS are illustrated in Table 2. The highest CBD plasma levels, ranging from 356 to 64 ng/ml (mean CBD level 175 ± 102 ng/ml), were related to Epidyolex administered at a mean dosage of 15 mg/kg/day. Linear regression analysis (Figure 1) showed a correlation between CBD concentrations measured on capillary blood sampled by VAMS did not differ from those measured by venous VAMS (*R* ^2^ > 0.98) and plasma from venipuncture (*R* ^2^ > 0.93).

**TABLE 2:** CBD therapeutic monitoring by VAMS and venipuncture including formulation and dose cannabis-based treatment.

	Patient 1	Patient 2	Patient 3	Patient 4	Patient 5
CBD plasma (ng/ml)	356	119	144	169	64
CBD venous VAMS (ng/ml)	447	163	141	190	72
CBD capillary VAMS (ng/ml)	405	153	112	122	52

CBD, cannabidiol; VAMS, volumetric absorptive microsampling.

## Discussion

TDM is often indispensable in the follow-up of epilepsy patients for the need of dose adjustments to optimize the clinical outcome (Kok and Fillet, 2018; Patsalos et al., 2018). VAMS devices are porous hydrophilic tips that enable an accurate collection of small blood volumes (Denniff and Spooner, 2014) avoiding the volumetric HCT bias and erythrocyte volume fraction bias associated with the non-volumetric dried blood spots approach (De Kesel et al., 2014; Denniff and Spooner, 2014; Spooner et al., 2015). Moreover, a significant advantage of this less invasive and easily reproducible procedure is that it limits the discomfort caused to patients in obtaining venous samples. However, to date, the TDM of medical cannabis has few validated analytical methods on plasma (Grauwiler et al., 2007; Aizpurua-Olaizola et al., 2017; Lomonaco et al., 2018; Pigliasco et al., 2020).

We used a new microsampling method for the determination of CBD blood levels in patients with drug-resistant epilepsy using VAMS, which had previously proven useful for quantitative measurement of several venous and capillary blood drugs, including first and third-generation antiepileptic drugs (Velghe and Stove, 2018; DʼUrso et al., 2019), antibiotics (Barco et al., 2017) and immunosuppressants (Koster et al., 2019).

Specifically, we aimed to evaluate the correspondence between the CBD levels detected in plasma and those measured using the VAMS technique, by pricking the patient’s finger. CBD concentrations that were taken from capillary blood by VAMS were not statistically different from those of venous blood obtained in the laboratory from the same device. Also, this statistical comparison proved to be valid between the results collected from VAMS microsampling and the CBD plasma levels obtained by venipuncture.

Several factors may influence the pharmacokinetics of CBD-related products used (Lucas et al., 2018; Birnbaum et al., 2019). In particular, CBD is related to a high potential of drug-drug interactions due to the influence on the activity of several enzymes involved in the metabolism of antiseizure medications, including cytochromes CYP2C and CYP3A, isoenzymes of CYP450. The known increase in plasma levels of N-desmethylclobazam (N-CLB), an active metabolite of clobazam, due to inhibition of the catalytic activity of CYP2C19 by CBD, is responsible for the most common dose-dependent adverse event in the clinical practice (Lattanzi et al., 2020) In this study, we did not methodically collect the plasma N-CLB levels in our patients treated with clobazam. Moreover, our study was not designed to monitor high intra- and inter-individual pharmacokinetic variability, although the implementation of the patient cohort could provide additional investigation material.

## Conclusion

VAMS device can be used as valuable support for patients with refractory epilepsy allowing control of CBD concentrations and dosage regulation, minimizing interindividual pharmacokinetic and pharmacodynamic problems, obtaining an effective personalized treatment and better control of therapeutic adherence. Our findings should be confirmed in further follow-up studies on larger series to identify a standardized match between the administered CBD dose and its detectable plasma concentration.

## Data Availability Statement

The raw data supporting the conclusions of this article will be made available by the authors, without undue reservation.

## Ethics statement

The studies involving human participants were reviewed and approved by CER Liguria. Written informed consent to participate in this study was provided by the participants’ legal guardian/next of kin.

## Author Contributions

All authors listed have made a substantial, direct, and intellectual contribution to the work and approved it for publication.

## Conflict of Interest

PS received advisory board fees from GW pharma and research grants from GW pharma and Enecta.

The remaining authors declare that the research was conducted in the absence of any commercial or financial relationships that could be construed as a potential conflict of interest.
